# Effects of Maxillary Protraction Techniques on Maxillofacial, Dental, and Soft Tissue Outcomes in Patients With Non‐Syndromic Unilateral Cleft Lip and Palate: A Systematic Review and Meta‐Analysis

**DOI:** 10.1111/ocr.70014

**Published:** 2025-08-08

**Authors:** Hamza Parvez Siddiqui, Anne Marie Kuijpers‐Jagtman, Karthik Sennimalai, Maria C. Meazzini, Madhanraj Selvaraj, Jitender Machawal

**Affiliations:** ^1^ Department of Orthodontics, Faculty of Dentistry, Sir John Walsh Research Institute University of Otago Dunedin New Zealand; ^2^ Department of Orthodontics University Medical Center Groningen, University of Groningen Groningen the Netherlands; ^3^ Department of Orthodontics and Dentofacial Orthopedics, School of Dental Medicine/Medical Faculty University of Bern Bern Switzerland; ^4^ Department of Orthodontics, Faculty of Dentistry Universitas Gadjah Mada Yogyakarta Indonesia; ^5^ Department of Orthodontics All India Institute of Medical Sciences Vijaypur Jammu India; ^6^ Department of Maxillo‐Facial Surgery, Smile House, Regional Center for CLP San Paolo e Carlo Hospital, University of Milano Milano Italy; ^7^ Consultant Orthodontist Puducherry India; ^8^ Central Hospital Asansol India

**Keywords:** cleft palate, expansion, facial growth, humans, maxillary protraction, orthodontics

## Abstract

**Objectives:**

To perform a quantitative assessment and best‐evidence synthesis of maxillary protraction techniques for unilateral cleft lip, alveolus, and palate (UCLAP), incorporating all available studies regardless of methodological quality.

**Materials and Methods:**

Studies on growing non‐syndromic UCLAP patients requiring maxillary protraction—against treated or untreated comparator groups—were identified across databases including PubMed, Embase, Scopus, Web of Science, EBSCOhost, Ovid MEDLINE, LILACS, Cochrane Library, and grey literature until April 23, 2025. Risk of bias for included studies was assessed using the Methodological Index for Non‐randomised Studies and the Cochrane Risk of Bias tool. A random effects model was applied using RevMan for quantitative analysis.

**Results:**

The search identified 1,892 articles, of which 36 met the inclusion criteria, categorised under four protraction methods: bone‐anchored, skeletally‐anchored facemask, facemask (FM) only, and intraoral springs, with or without expansion. Quantitative synthesis was feasible only for the FM group. Compared to no‐protraction UCLAP (NP‐UCLAP), Protraction group (P‐UCLAP) showed a significant short‐term increase in ANB angle (MD = 4.34; 95% CI: 3.65–5.03; *p* < 0.00001; *I* ^2^ = 73%). When compared to treated non‐cleft individuals, FM therapy also showed a significant ANB increase (MD = 0.86; 95% CI: 0.42–1.29; *p* = 0.0001; *χ* ^2^ = 4.65; *I* ^2^ = 35%).

**Conclusion:**

FM therapy shows short‐term benefits from low‐quality studies for UCLAP maxillofacial growth and future studies might not be necessary as supported by sequential analysis. Evidence for skeletally anchored methods and addition of expansion to any protraction method remains weak and rigorous, long‐term studies are needed.

## 1 Introduction

Cleft lip and/or palate (CL/P) with an estimated incidence of 1:700 per live birth appears to be the commonest congenital craniofacial defect caused by incomplete fusion of facial prominences during the fourth to tenth week of gestation [1, 2]. The aetiology of cleft lip and palate (CLP), however, is still not fully understood, but both environmental and genetic factors are assumed to be responsible [3, 4]. Complete unilateral cleft lip, alveolus and palate (UCLAP) accounts for about 70% of all orofacial clefts [5]. These patients are characterised by a deficient nasomaxillary complex due to intrinsic, functional and iatrogenic factors such as scarring from primary surgeries, along with reduced transverse, sagittal and vertical dimensions of the nasal and facial structures [6, 7, 8]. Severe maxillary retrusion in school‐aged children and adolescents with UCLAP poses a significant risk for personal, social and psychological morbidity due to its impact on facial aesthetics and speech [9, 10].

To address these concerns, early orthopaedic correction has been explored as a potential solution. One commonly used approach is early maxillary protraction with a facemask (FM), which applies extraoral forces to improve skeletal discrepancies in UCLAP patients. However, this therapy is known to be associated with dentoalveolar effects such as severe labial tipping of the maxillary incisors, extrusion of the maxillary molars, clockwise rotations of the mandibular plane, bite opening, and increase in the lower facial height [10, 11, 12]. Additionally, wearing a very visible facial device makes compliance a challenge and frequent clinical adjustments add to the burden of care [13].

To address these limitations, modified surgical miniplates have been introduced, both with and without facemask therapy. These provide skeletal anchorage, enhancing orthopaedic effects and reducing dental side effects [14, 15, 16]. A more widely recognised approach that has gained significant attention is the bone‐anchored maxillary protraction (BAMP) protocol developed by De Clerck et al. This method involves placing miniplates in the maxilla and mandible and applying continuous traction using Class III intermaxillary elastics for 24 h daily [17]. The advantages of this protocol, particularly the improved patient compliance, reassure us about the effectiveness of the BAMP protocol and its potential to extend treatment duration.

While early orthopaedic traction from aforesaid techniques promises a treatment strategy, selecting the most suitable modality remains challenging. The literature offers numerous systematic reviews investigating the effects of maxillary protraction using FM, bone‐anchored maxillary protraction (BAMP), mini‐screws or various combinations of these methods in non‐cleft patients [18, 19]. However, studies on the dentoskeletal effects of maxillary protraction in patients with unilateral cleft lip alveolus and palate (UCLAP) are more limited. Previously conducted meta‐analyses have addressed these effects; however, a significant limitation of traditional meta‐analyses is their focus on reliability and replicability at the expense of a deeper understanding of the issues [12, 20]. This underscores the need for further research, inviting the scientific community to contribute to the refinement of treatment protocols and optimisation of patient outcomes for those with UCLAP.

In the current review, we conducted a quantitative assessment along with best‐evidence synthesis, incorporating all available studies, irrespective of methodological quality. This approach minimises reviewer bias in determining whether a study is “good” or “bad” based solely on its methodology. To the best of our knowledge, this review is the first of its kind to provide a comprehensive evaluation of methodological quality, consistency of findings, and available outcomes for maxillary protraction in patients with UCLAP, ensuring a more effective and informed approach to clinical management while considering the burden of care.

## 2 Material and Methods

### 2.1 Protocol and Registration

The systematic review was conducted in accordance with the Cochrane Handbook for the Systematic Review of Interventions [21] and reporting was done in accordance with the Preferred Reporting Items for Systematic Reviews and Meta‐Analyses (PRISMA) [22]. The review was registered in the PROSPERO database (number CRD42021229018) and can be accessed.

### 2.2 Eligibility Criteria

The research question was formulated using the PICOS structure (Population, Intervention, Comparator, Outcome, Study design):

#### 2.2.1 Participant

Non‐syndromic growing patients with UCLAP requiring maxillary protraction of any sex and ethnicity. Syndromic patients or patients with an orofacial cleft other than UCLAP were excluded.

#### 2.2.2 Intervention

Studies with at least one group of patients with UCLAP treated with FM or skeletally anchored protraction (BAMP or FM‐Miniplate) or spring‐assisted. Studies relating to orthognathic surgeries were excluded.

#### 2.2.3 Comparator

Varying types of maxillary protraction in a cleft or non‐cleft sample, or untreated controls.

#### 2.2.4 Primary Outcome

Maxillofacial growth assessed 2‐dimensionally or 3‐dimensionally on lateral cephalograms or cone beam computerised tomography scans (CBCT), respectively, including skeletal and dento‐alveolar soft tissue measurements.

#### 2.2.5 Secondary Outcome

Occlusal relationship assessed with the Great Ormond Street London and Oslo (GOSLON) yardstick.

#### 2.2.6 Study Design

Randomised controlled trials (RCTs), controlled clinical trials (CCT), prospective cohort studies, retrospective studies and intervention studies evaluating the effectiveness of maxillary protraction involving human subjects were included. Systematic reviews, meta‐analyses, case series, case reports, animal studies, studies using finite element analysis and expert opinions were excluded.

### 2.3 Information Sources and Search Strategy

Electronic databases the Cochrane Database of Systematic Reviews, Cochrane Central Register of Controlled Trials (CENTRAL), PubMed, Scopus, Embase, Web of Science, Google Scholar, Ovid MEDLINE, EBSCOhost and LILACS were searched from their inception until April 23, 2025. Ongoing and unpublished studies were searched using ClinicalTrials.gov (http://www.ClinicalTrials.gov), and the ISRCTN registry (http://www.controlled‐trials.com). There were no limits for language or date of publication. Details of the search strategy are given in Table S1. In addition, to ensure full coverage, the reference lists of the included literature, first 200 articles of the Google Scholar and grey literature were also examined [23]. Wherever the data was not available for the study or was suspected to be an extension of the previously published literature, corresponding authors were tried to contact via one or two mails.

### 2.4 Data Collection & Data Items

A data extraction form was designed and piloted. After the form was finalised, two reviewers (HS and JM) independently performed the data extraction. The details that were extracted from each study included: study setting and design, characteristics of participants in the experimental and control group, intervention performed, details of the appliances, duration of use of the appliances, force design and observation time, and, lastly, the following nine angular cephalometric parameters were studied as outcomes: Skeletal sagittal (SNA/S–N–ss, SNB/S–N–sm, ANB/ss–N–sm), dentoalveolar (IMPA, SN–MP, U1–SN, L1‐GoGn), Skeletal vertical (GoGn‐Sn), Nasolabial angle (PRN‐SS‐SN); however, meta‐analysis was selected to be sought on commonly available parameters among these studies. Additionally, the GOSLON index was looked at as a secondary outcome. Linear cephalometric parameters were not included to avoid inherent errors of magnification. On various occasions, similar cephalometric measurements were reported by the authors. All equivalent names of the same measurement were grouped together, and only one term was used throughout the article. Where data was not available, corresponding authors were contacted for the same.

### 2.5 Risk of Bias in Individual Studies

Two reviewers (HS and KS) independently assessed the risk of bias in the included studies using the Methodological Index for Non‐randomised Studies (MINORS) scale for nonrandomised studies [24] and the Cochrane Risk of Bias (RoB 2) tool for randomised studies [25]. The scoring for MINORS was done on 12 items wherein each item was scored as 0 (not reported), 1 (reported but inadequate), or 2 (reported and adequate) with a global ideal score of 24 for comparative studies. For this review, cutoff points were taken as ≤ 14, 15–21, and 22–24 for poor, moderate and high‐quality studies, respectively. Any disagreement between the ratings of the reviewers was resolved after a discussion with a third reviewer (MS).

### 2.6 Summary Measures

For descriptive continuous data mean, standard deviation (SD), sample size, weighted mean differences and 95% confidence intervals were calculated. A random effects model was applied using RevMan version 5.3.4 (Cochrane Collaboration) to adequately account for the different treatment protocols, appliances, patient characteristics, and measurement techniques. Statistical heterogeneity between studies was checked by Cochran *X* ^2^‐based *Q*‐test and the *I* ^2^ static [26]. An inconsistency score (*I* ^2^) was computed, where an *I* ^2^ value falling in the range of 30%–60% may represent moderate heterogeneity, and an *I* ^2^ value ranging from 75% to 100% signified a considerable degree of heterogeneity. A two‐tailed *p* value of 0.05 was set as point of significance for hypothesis testing, except for the test of heterogeneity and publication bias, where a *p* value of 0.01 was applied due to low power.

### 2.7 Best Evidence Synthesis

A thorough overview of all findings comparing interventions of maxillary protraction using different treatment techniques and different outcome measures based on cephalometric parameters and GOSLON outcome was prepared. However, if the data gathered from the literature were insufficient to be included in a meta‐analysis, the best evidence synthesis (BES) approach was used [27]. This method considers the methodological quality and consistency of outcomes in terms of numbers, along with the results withdrawn from the included studies.

The studies were graded as follows:

1. Strong evidence, provided by ≥ 2 high‐quality studies (HQS) which can be meta‐analysed;
2. Moderate evidence, provided by consistent findings in multiple HQS and low‐quality studies (LQS) when evidence was sufficient to be pooled together, or in > 2 consistent low‐quality studies to be pooled;
3. Insufficient evidence, when only 1 HQS was available, or findings were inconsistent in multiple LQS and data cannot be pooled.

The parameters were considered ‘consisten’ if at least 75% of studies using the same PICO produced similar results and were defined based on significance (*p* < 0.05). Whenever the P value was not given, this was specifically indicated. If ≥ 2 studies were of high methodological quality, we ignored the studies of low methodological quality. If all the HQS and LQS were present at the same time and suitable to be pooled together, they were included in the meta‐analysis without excluding any of them.

## 3 Results

### 3.1 Study Selection

**FIGURE 1:**
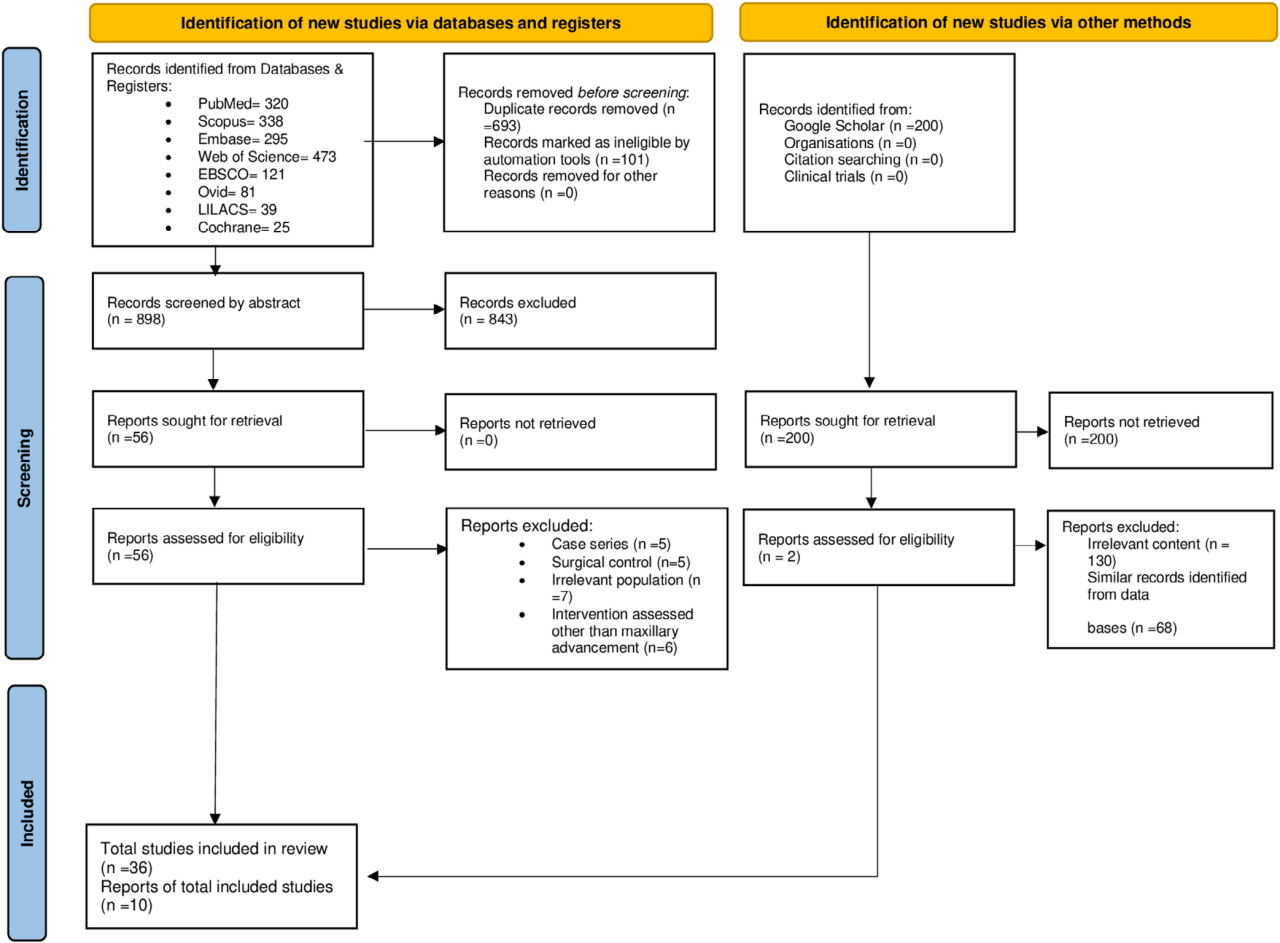
PRISMA flow diagram depicting the study selection process.

The PRISMA flow diagram is shown in Figure 1. The search strategy yielded a total of 1892 articles from different databases in the first search. No additional articles were found from the manual search. Following the removal of duplicates, 898 articles were screened for eligibility. After reviewing the titles and abstracts, 58 articles were selected and subjected to complete text evaluation. Finally, 36 articles met the eligibility criteria, including 14 retrospective comparative, two RCT, two cohort, and 18 prospective comparative studies.

Table 1 shows the demographics and characteristics of the included studies. The 36 selected studies were published in the English language between 1993 and 2025. The participants' age at the start of treatment ranged from 5 to 13 years and had their cleft repaired. The details of the primary surgeries were only available in 10 studies [10, 28, 29, 30, 31, 32, 33, 34, 35, 36].

**TABLE 1:** Characteristics of the included studies.

First author year	Clinical setting	Study design	Sample size	Sex	Mean age	Primary surgeries
Country			Exp/Control	Exp/Control	Exp/Control	
Tindlund 1993 Bergen, Norway	University of Bergen	Retros	63/24	43 M, 20 F/19 M, 5 F	6 y 10 mo	L + P + anterior part of secondary palate at 3 mo
Tindlund 1993 Bergen, Norway	University of Bergen	Retros	63/24	NR	6 y 10 mo/7 y 0 mo	L + P + anterior part of secondary palate at 3 mo
Buschang 1994 Texas, USA	Baylor College of Dentistry	Retros	21/21	7 M, 14F/7 M, 14F	7.3 ± 1.4 y	NR
Chen 1996 Hong Kong	University of Hong Kong	Pros	10/10	20 M	9.67 y	L = 3 mo; P = 18 mo
So 1996 Hong Kong	University of Hong Kong	Pros	10/10	20 F	10.57 ± 1.31 y	L = 3 mo; P = 18 mo
Chen 1997 Hong Kong	University of Hong Kong	Pros	10/10	20 M	9.67 y	L = 3 mo; P = 18 mo
Liou 2005 Taiwan	Chang Gung memorial hospital	Pros	16/10	8 M, 8 F/4 M, 6 F	9–12 y/9–12 y	NR
Ramadan 2008 Cairo, Egypt	Suez Canal University	Pros	10/10	20 M	5–8 y; left sided cleft/5–8 y	NR
Jia 2008 Beijing, China	School of Stomatology, Peking University	Pros	18/18	36 M	9.54 ± 1.21 y	NR
Gustavo 2009 Brazil	University of Rio Grande do Sul	Pros	10/10	4 M, 6 F/5 M, 5 F	10.4 ± 2.62 y/10.4 ± 2.62 y	NR
Sade Hoefert 2010 Tübingen, Germany	Eberhard Karl University	Pros	8/21	5 M, 3 F/8 M 13 F	5.6 ± 0.82 y/5.3 ± 0.48 y	NR
Dogan 2012 Izmir, Turkey	Ege University	Retros	20/20	12 M, 8 F/10 M, 10 F	male = 8.70 ± 2.64 y female = 8.69 ± 1.64 y	NR
Ahn 2012 Seoul, South Korea	Seoul National University	Retros	15/15	13 M, 2 F/15 M	11.42 ± 1.86 y/10.98 ± 1.92 y	L = Milard's‐3 mo P = Furlow's double opposing one stage Z‐plasty—12–18 mo
Farhani 2014 Los Angeles, USA	Children's Hospital	Retros	18/17	10 M, 8 F/8 M, 9 F	13.4 ± 0.45 y/13.5 ± 0.44 y	L = Milard's P = greater palatine flap
Singla 2014 Chandigarh, India	Sanjay Gandhi post‐graduate Medical Research institute, India	Pros	19/5	14 M, 5 F/4 M, 1 F	9.36 ± 2.89 y/8.25 ± 2.25 y	NR
Fu 2016 Beijing, China	School and Hospital of Stomatology, Peking University	Cohort	18/14	13 M, 5 F/9 M, 5 F	10.4 ± 1.3 y/9.6 ± 1.7 y	NR
Tome 2016 Osaka, Japan	Osaka University	Retros	19/19	9 M,10 F/12 M, 7 F	7 y 2 mo/7 y 5 mo	E: Early two stage Furlow C: Push back = 12–18 mo
Kecik 2017 Istanbul, Turkey	Baskent University	Retros	23/26	11 M, 12 F/12 M,14 F	8.3 ± 2.4 y/8.1 ± 2.5 y	NR
Yatabe 2017 Sao Paulo, Brazil	Hospital for Rehabilitation of Craniofacial Anomalies	Pros	20/24	NR	11.8 y/11.9 y	NR
Zhang 2018 Beijing, China	School and Hospital of Stomatology, Peking University	Pros	36/18	54 M	9.98 ± 1.10 y/9.76 ± 1.43 y	NR
Singla 2018 Chandigarh, India	Postgraduate Research Institute University	Pros	17/5	13 M, 4 F/4 M, 1 F	9.28 ± 2.78 y/8.25 ± 2.25 y	NR
Hassan 2018 Hong Kong	University of Hong Kong	Retros	14/24	6 M, 8 F/14 M, 10 F	11.3 ± 0.6 y	NR
Meazzini 2019 Milano, Italy	San Paolo Hospital, University of Milan	Retros	26/12	16F,10M	11.7 y/11.3 y	NR
Zhang 2019 Beijing, China	Peking University Hospital of Stomatology	Retros	12/7	7 M, 5 F/5 M, 2 F	10.5 ± 1.3 y/11.1 ± 0.6 y	NR
Ren 2019 Groningen, Netherlands	University Medical Center Groningen	Pros	18/31	12 M,6 F/(8 M, 2F) (10 M, 11 F) (6 M, 4 F)	11.3 ± 0.6 y/(11.6 ± 1.4) y (11.8 ± 0.6) y (11.5 ± 1.1) y	NR
Faco 2019, Sao Paulo, Brazil	Hospital for Rehabilitation of Craniofacial Anomalies	Pros	27/23	19 M, 8 F/17 M, 6 F	11.7 y/11.5 y	NR
Ozawa 2020 Sao Paulo, Brazil	Hospital for Rehabilitation of Craniofacial Anomalies	Retros	34/20	NR	Between 9 y and 12 y	L = Milard's: 3 mo P = Von Langenback/Furlow's double 9–18 mo
Dogan 2020 Izmir, Turkey	Ege University, Department of Orthodontics	Pros	15/15	8 M, 7 M/7 M, 8 F	10.07 ± 2.43 y	NR
Elabassy 2020 Cairo, Egypt	Cleft Care Center Ain Shams University	Pros	14/14	10 M, 4 F/6 M, 8 F	10.3 6 ± 0.9 y/11.3 6 ± 1.4 y	NR
Lin 2021 Hong Kong	Peking University Hospital of Stomatology	Pros	26/26	18 M, 8 F (19 left sided cleft; 7 right sided cleft)/14 M, 12 F	10.32 ± 1.29 y/9.82 ± 1.03 y	NR
Steegman 2021 Groningen, Netherlands	University Medical Center Groningen	Cohort	19/17	NR	11.4 ± 0.7	NR
Hashem 2021 Egypt	Minia University	Retros	15/15	8 M, 7 F/7 M, 8 F	8.3 ± 1 y/8.5 ± 1.1 y	NR
Yu 2021 Seoul, Korea	Seoul National University Children's Hospital	Retros	33/20	24 M, 9 F/16 M, 4 F	10.27 ± 0.63 y/10.75 ± 0.97 y	L = Milard's‐3 mo P = Furlow's double opposing one stage Z‐plasty—12–18 mo
Gupta 2022 India	Siddhpur Dental College and Hospital	Pros	17/5	12 M, 9 F/5 M, 2 F	11.23 ± 3.56 y/7.95 ± 3.56 y	NR
S Dutta 2024 Faridabad, India	Manav Rachna Dental College	RCT	15/15	19 M, 11 F	9.40 ± 2.50 y	NR

Studies	Intervention experimental/control	Expansion	Protraction protocol	Duration of treatment	Bone graft with timing	Control group characteristics	Retainer
Tindlund 1993	E: FM‐Delaire C: FM‐Delaire	QH	F = 350 g per side DF = 15° to the OP W = Night time for 11 h	E: 12 mo C: 15 mo	NR	BCLAP	NR
Tindlund 1993	E: FM‐Delaire C: FM‐Delaire	QH	F = 350 g per side DF = 15° to the OP W=Night time for 11 h	E: 12 mo C: 15 mo	NR	BCLAP	NR
Buschang 1994	E: FM‐Petit C: No Tx	RME (0.2 mm/day)	F = 350 g per side DF = NR W = 12–14 h a day	1.2 y	NR	Non‐cleft	NR
Chen 1996	E: FM‐Tübinger C: No Tx	NA	F = 400–500 g each side DF = 10° to the OP W = 12‐14 h/day	7.8 mo	NR	UCLAP	Reverse Activator after 5 mm positive overjet
So 1996	E: Reverse Headgear C: No Tx	NA	F = 450–500 g each side DF = 10° to the OP WT = 12–14 h/day	9.7 mo	NR	UCLAP	NR
Chen 1997	E: FM‐Tübinger C: No Tx	NA	F = 400–500 g each side DF = 10° to the OP W = 12‐14 h/day	7.8 mo	NR	UCLAP	Reverse Activator after 5 mm positive overjet
Liou 2005	E: Protraction spring C: Protraction spring	E: Alt‐RAMEC (9 weeks) C: RME (1 week)	F = 400–500 g DF = horizontal and upward force	5 mo	NR	UCLAP	NR
Ramadan 2008	E: FM C: FM	NR	F = 250–300 g each side DF = horizontal and upward force WT = 18 h/d until correction of the anterior crossbite.	9 mo	NR	Class III	NR
Jia 2008	E: RH C: RH	NR	F = 400–500 g each side DF = 10° to the OP WT = 12 h/day, till 2 mm of positive overjet	9.2 ± 1.6 mo	NR	Non‐cleft Class III with anterior crossbite	NR
Gustavo 2009	E: FM‐Petit C: FM‐Petit	E: Alt‐RAMEC (7 weeks) C: RME (1 week)	F = 400–500 g each side DF = 30° to the OP WT = NR	6 mo	NR	UCLAP	NR
Sade Hoefert 2010	E: FM‐Delaire C: FM‐Delaire	RME	F = NR DF = NR WT = NR	8.2 (6.9–9.8) mo	NR	Isolated cleft palate, Class III, Class I	NR
Dogan 2012	E: FM‐Delaire C: No Tx	RME	F = 800 g DF = 25° to the OP WT = 16 h/day	7 mo 5 d	NR	Non cleft Class I	NR
Ahn 2012	E: MxP + FM (Petit) C: MxP + FM (Petit)	NA	F = > 500 g each side DF = 30° to the OP WT = 12–14 h/day	2.36 y	NR	BCLAP	NR
Farhani 2014	E: FM C: No Tx	Alt‐RAMEC	F = NR DF = 30° t the OP WT = NR	4.6 mo	Alveolar bone grafts before participation in the study	UCLAP with Class III	CIII elastics along during orthodontic Tx
Singla 2014	E: FM‐Delaire C: No Tx	NA	F = 420–480 g on each side DF = 15° to 30° to the OP WT = 16 to 18 h per day.	11.71 ± 3.39 mo	NR	UCLAP	NR
Fu 2016	E: FM‐Tubinger C: No Tx	NA	F = 450–500 g each side DF = 20°–30° to the OP WT = 12 h/day till 2 mm positive overjet	8 mo	NR	UCLAP	NR
Tome 2016	E: FM C: FM	E: QH C: QH	F = NR DF = NR WT = NR	E: 27.3 mo C: 29.4 mo	NR	UCLAP	NR
Kecik 2017	E: FM C: FM	NR	F = 450–500 g each side DF = 30° to the OP WT = 12–14 h/day, till 2 mm of positive overjet	0.8 y	NR	Class III	NR
Yatabe 2017	E: BAMP C: BAMP	NR	F = initially 150 g, increased gradually to 200–250 g; change elastics twice a day DF = NR WT = NR	18/12 mo	SABG 3 mo prior to miniplate placement	Class III	NR
Zhang 2018	E: FM‐Tubinger C: No Tx	NA	F = 450–500 g each side DF = 30° to the OP WT = 12–14 h/day, till 2 mm of positive overjet	12 mo	SABG prior to protraction	UCLAP	NR
Singla 2018	E: FM‐Delaire C: No Tx	NR	F = 420–480 g on each side DF = 15°–30° to the OP WT = 16–18 h/day.	11.62 mo	NR	UCLAP	NR
Hassan 2018	E: FM C: No Tx	NR	F = 400 g on each side DF = NR WT = 12 h/day	9–12 mo	SABG prior to protraction	UCLAP	NR
Meazzini 2019	E: TAD assisted protraction C: No Tx	Alt‐RAMEC in experimental only	F = springs 300 g per side DF = NR WT = 24 h/day	5–8 mo	NR	UCLAP	NR
Zhang 2019	E:FM C:FM	NR	F = 400 g on each side DF = from the canine region to the OP WT = 12 h/day	1.8 ± 0.8 y	SABG before protraction	UCLAP	NR
Ren 2019	E: BAMP C: No Tx	NR	F = initially 150 g, increased gradually to 200–250 g; change elastics once a day DF = NR WT = 24 h/day	18 mo	SABG before protraction	UG non‐cleft‐ 10; UG‐cleft‐ 11; UG‐Cleft‐10	NR
Faco 2019	E: BAMP C: No Tx	RME during mixed dentition	F = initially 150 g, increased gradually to 200–250 g; change elastics twice a day DF = NR WT = 24 h/day	18 mo	SABG at least 6 months before study onset	UCLAP	NR
Ozawa 2020	E:FM C:FM	RME	F = 407 g DF = NR WT = 18 h/day	9 mo	No SABG before FM therapy	UCLAP	NR
Dogan 2020	E:FM‐Delaire C:FM‐Delaire	Alt‐RAMEC in experimental only	F = 600 g each side DF = 30° to the OP WT = 12–14 h/day, till 2 mm of positive overjet	4.7 mo	NR	UCLAP	NR
Elabassy 2020	E: BAMP C: BAMP	Fan shaped expander in E only	F = initially 150 g, increased gradually to 200–250 g DF = NR WT = 24 h/day	9 mo	NR	UCLAP	NR
Lin 2021	E:FM‐Petit C:FM‐Petit	NA	F = 450–500 g each side DF = 20°–30° to the OP WT = 12 h/day till positive overjet	E:18.44 ± 4.16 mo C: 12.46 ± 4.03 mo	SABG before treatment	Class III	NR
Steegman 2021	E: BAMP C: No Tx	NR	F = initially150 g, increased gradually to 200–250 g; change elastics once a day DF = NR WT = 24 h/day	1.5 y; follow up 3.5 year	SABG priorly	UCLAP	NR
Hashem 2021	E:FM‐Petit C: No Tx	RME	F = NR DF = 30° to the OP WT = 14 h/day till 1 mm positive overjet	1.6 ± 0.4 y	NR	Non‐cleft Class I	NR
Yu 2021	E:Petit FM + MP C: Petit FM + MP	NR	F = 500 g each side DF = 20°–30° to the OP WT = 12‐14 h/day	NR	SABG before protraction	UCLAP	NR
Gupta 2022 I	E:FM‐Delaire C: No Tx	NR	F = 440–490 g each side DF = NR WT = 14‐18 h/day	12.22/13.56 mo	NR	UCLAP	NR
S Dutta 2024	E: Petit FM + MP C: Petit FM	Alt‐Ramec in control group	F = 400 g each side DF = 20°–30° to the OP WT = 15–16 h/day	9.3 ± 2.1 mo	No SABG before FM therapy	UCLAP	NR

Abbreviations: Alt‐RAMEC, alternate rapid maxillary expansion and contraction; BAMP, bone anchored maxillary protraction; BCLAP, bilateral cleft lip, alveolus, and palate; C, control; d, days; DF, direction of force; E, experimental; F, force; FM, face mask; L, lip; mo, month; MP, miniplate; NA, not applicable; NR, not reported; OP, occlusal plane; P, palate; QH, quad helix; SABG, secondary alveolar bone grafting; Tx, Treatment; UCLAP, unilateral cleft lip, alveolus, and palate; UG, Untreated Group; WT, wear time; y, year.

Two studies were conducted with duplicate participants as a part of soft tissue evaluation [31, 34] of a previously reported skeletal evaluation [30, 33]. These studies investigated the effect of maxillary protraction by FM or BAMP or protraction springs or Temporary Anchorage Devices (TAD), assisted with or without maxillary expansion.

The duration of the follow‐up varied from 5 months to 4 years with a force range of 300–600 g per side for 12–16 h per day for FM therapy except for one where higher forces of 600 g per side were used [37] and up to 250 g with BAMP.

With regard to the control group, 13 studies compared Protraction‐UCLAP (P‐UCLAP) versus No‐protraction (NP‐UCLAP) [10, 29, 30, 31, 38, 39, 40, 41, 42, 43, 44, 45, 46, 47], three with BCLP [28, 33, 34] two with separate groups of cleft and non‐cleft [48, 49], three studies with untreated non‐cleft [11, 38, 50], five studies with treated non‐cleft [51, 52, 53, 54, 55], while the remaining 10 compared with P‐UCLAP [32, 35, 36, 37, 56, 57, 58, 59, 60, 61, 62]. Hence, to homogenise the results, quantitative synthesis of those studies was conducted which compared P‐UCLAP with NP‐UCLAP (BAMP + FM) that included eight studies and P‐UCLAP versus treated non‐cleft which consists of three studies.

Among the included studies of the miniplate assisted protraction, all the participants had undergone bone grafting procedures prior to the commencement of the study along with prospective sample size calculation except one [60]. In contrast, only five studies using FM assisted protraction reported secondary alveolar bone grafting (SABG) done prior to protraction [29, 42, 53, 57, 63]. Table 1 shows details of the intervention used in the control and experimental groups. For the convenience of readers, we presented our results under different sections of BAMP, FMMP, FM, and protraction spring therapy.

### 3.2 Results BAMP Therapy

All the included studies were conducted recently between 2017 and 2025. All studies had a UCLAP control group except one [55]. Except for one study [60] which used custom‐made surgical miniplates from biocompatible alloy grade 2 in the upper and lower arch, all other studies [39, 46, 48] used Bollard miniplate as described elsewhere [17]. The site of insertion was the same across all the studies being placed in the maxilla at the infrazygomatic crest and between the lateral incisor and canine in the lower arch. Maxillary protraction was initiated 3 weeks post‐miniplate placement, starting initially with a load of 150 g and gradually increasing to 250 g after 2 months. The participants were instructed to apply 24‐h traction with elastics and change it once [46, 48] or twice daily [39, 55]. None of the participants had undergone any treatment for transverse changes except in one study, which aimed to study the effect of BAMP on dentoskeletal parameters assisted with and without rapid maxillary expansion (RME) [60]. The subjects in the study group were treated for 18 months in all the studies except for one where the treatment duration was about 9 months [60] and CBCT‐derived lateral cephalogram was analysed after the achievement of positive overjet. Skeletal and dento‐alveolar angular measurements were reported by all except one [55] which reported results as three‐dimensional displacement of maxillary landmarks.

No meta‐analysis could be performed as data from only two studies [39, 48] was found homogeneous under this group. Only one study reported longer follow‐up [46]. However, it was not included as part of the formal meta‐analysis since it was suspected to have the same cohort of patients based on a similar trial number as used in another study [48]. All studies did report a formal sample size calculation. No information about the retention protocol was available in any of the studies in this group.

### 3.3 Results Miniplate Assisted FM (FMMP) Therapy

Three studies were found under this category. Two studies had UCLAP as their control group [36, 61], while one had BCLP control [28]. L‐shaped plates were placed in the infrazygomatic crest by raising a mucoperiosteal flap adapted to the surface and exposing it in the area between the canine and 1st premolar region. All the studies employed a Petit face mask for maxillary protraction assisted by mini plates in experimental and control groups with similar force vectors and duration of wear per day. However, there was a significant variation in treatment duration among the included studies in this group. One study had approximately nine months of protraction [61], while the other had over two years [28] and 4 years [36], respectively. The details of primary surgeries were available in two studies [28, 36] which were Millard's rotational and advancement flap for cheiloplasty 3–5 months after birth and Furlow's double opposing Z‐plasty for one‐stage palatography, 12–18 months after birth. Due to control group heterogeneity, no quantitative analysis was possible.

### 3.4 Results FM Therapy

Twenty‐seven studies were included under this category, each contributing to our understanding of maxillary protraction effects. Four studies [31, 34, 49, 50] reported only soft tissue effects; two [32, 42] reported changes in the GOSLON Yardstick Index [64], and the remaining reported changes in dentoskeletal parameters during FM assisted maxillary protraction. Except for three studies where participants were put on reverse activator in two [30, 31] and Class III elastics in another [29] on retention, no other studies reported any retention protocol. There was a wide variation in terms of maxillary expansion methods, as three studies [33, 34, 35] reported expansion by quad helix; five studies [11, 32, 38, 49, 50] used RME; three studies [29, 37, 62] employed Alt‐RAMEC; no expansion in [10, 30, 31, 40, 41, 44, 63] while expansion remained unclear in the rest of the included studies. One study compared dentoskeletal effects of Alt‐RAMEC assisted FM with sagittal TransForce appliance [62].

### 3.5 Quantitative Synthesis FM Therapy

**FIGURE 2:**
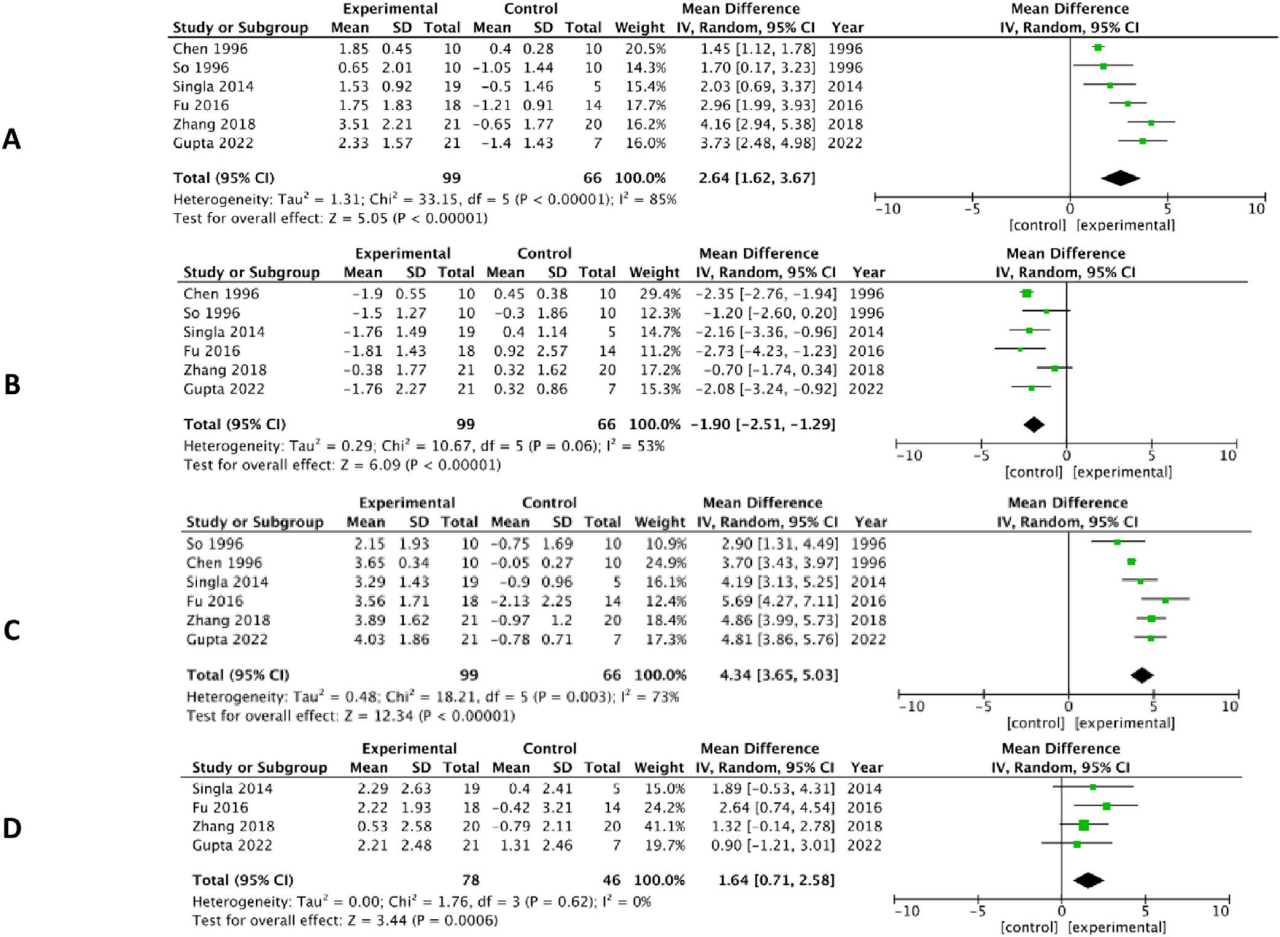
Forest plot using random‐effects model for comparing P‐UCLAP versus NP‐UCLAP (A) SNA, (B) SNB, (C) ANB and (D) vertical.

The results of quantitative analyses of conventional FM therapy were compiled as two groups. Group A included six studies [10, 30, 40, 41, 44, 47] where P‐UCLAP underwent maxillary protraction with FM without expansion and NP‐UCLAP participants that served as control. None of the participants had abnormal mandibular displacement. Additionally, one study compared maxillary protraction in grafted with non‐grafted UCLAP subjects and with untreated controls, but the results of the non‐grafted group were subjected to meta‐analysis. Moreover, as there were two studies (2014 and 2018) by Singla and co‐workers from the same centre (Post‐graduate Institute, Chandigarh, India), we only took one study (2014) owing to similar age groups and control in both studies. The results from these studies were quantified to SNA, SNB, ANB, Vertical skeletal, on lateral cephalograms (Figure 2). The included articles [10, 30, 40, 41, 44, 63] found a statistically significant increase in SNA (MD = 2.64; 95% CI: 1.62, 3.67; *p* < 0.00001; *χ* ^2^ = 36.88; *I* ^2^ = 85%) and ANB (MD = 4.34; 95% CI: 3.65, 5.03; *p* < 0.00001; *χ* ^2^ = 18.21; *I* ^2^ = 73%). There was a significant decrease in SNB (MD = −1.90; 95% CI: −2.51, −1.29; *p* < 0.00001; *χ* ^2^ = 10.67; *I* ^2^ = 53%) of the experimental group indicating a prominent chin‐cup effect. However, FM therapy was found to increase the vertical parameter (MD = 1.64; 95% CI: 0.71, 2.58; *p* < 0.0001; *χ* ^2^ = 1.76; *I* ^2^ = 0%) of the experimental group [40, 41, 44, 63].

**FIGURE 3:**
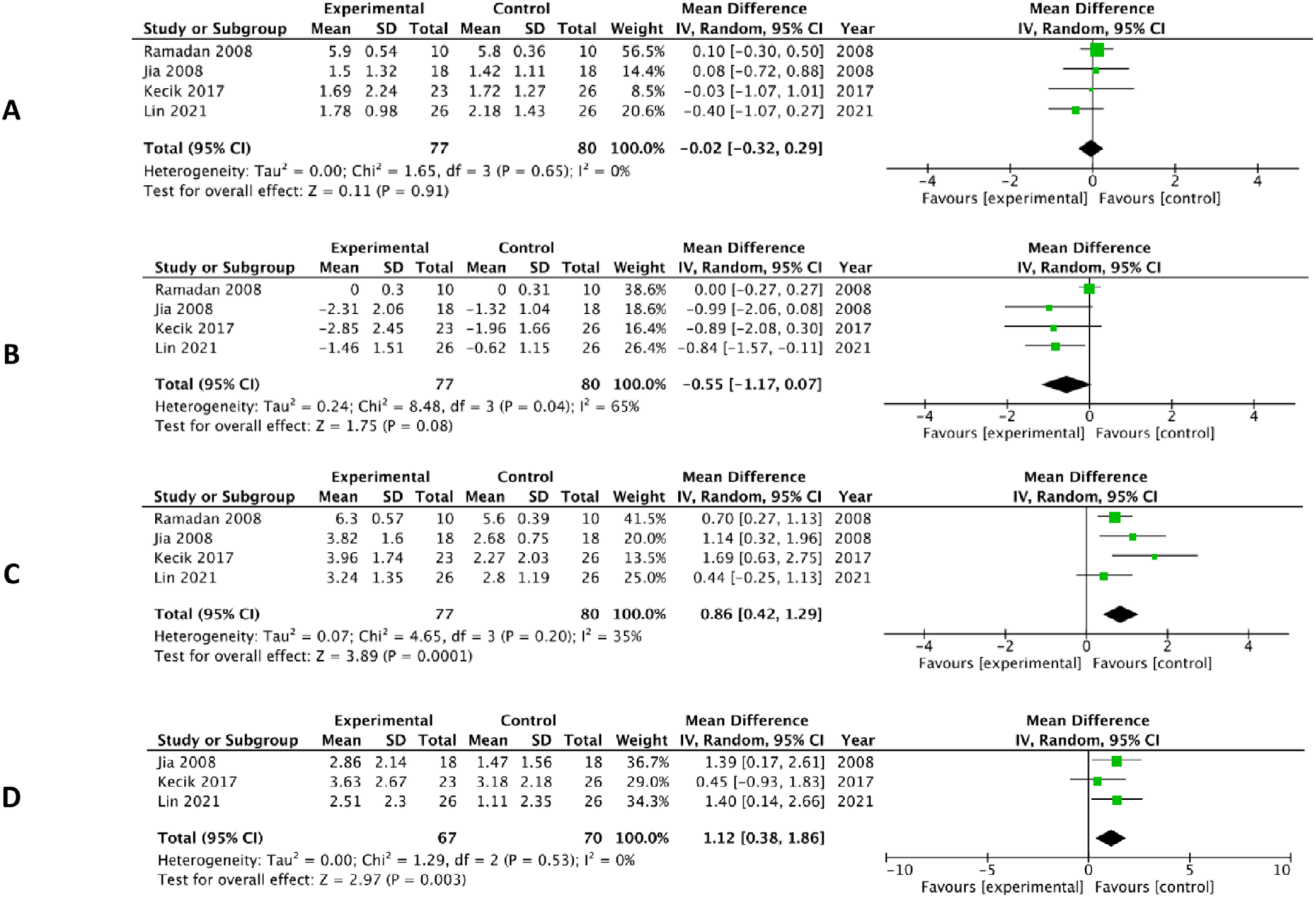
Forest plot using random‐effects model for comparing P‐UCLAP versus Class III (A) SNA, (B) SNB, (C) ANB and (D) vertical.

On the other side, Group B comprised four studies [51, 52, 53, 54] where P‐UCLAP groups were compared with treated Class III patients that underwent FM therapy. The results from these studies were meta‐analysed for SNA, SNB, ANB, Vertical skeletal, upper incisor (U1‐SN) and lower incisor (L1‐GoGn). No significant differences were seen between the two groups for maxillary angular and vertical changes that is, SNA (MD = −0.02; 95% CI: −0.32, 0.29; *p* = 0.91; *χ* ^2^ = 1.65; *I* ^2^ = 0%) and (MD = −0.11; 95% CI: −0.78, 0.56; *p* = 0.75; *χ* ^2^ = 0.0.03; *I* ^2^ = 0%) respectively but SNB was found to decrease more in the UCLP group than the CIII group (MD = −0.55; 95% CI: −1.17, −0.07; *p* = 0.008; *χ* ^2^ = 8.48; *I* ^2^ = 65%) after FM therapy. ANB changes marginally seemed to favour the UCLAP group as compared to the Class III group (MD = 0.86; 95% CI: 0.42, 1.29; *p* = 0.0001; *χ* ^2^ = 4.65; *I* ^2^ = 35%) (Figure 3). No changes were observed in dental parameters; U1‐SN (MD = 0.79; 95% CI: −1.60, 3.19; *p* = 0.52; *χ* ^2^ = 7.78; *I* ^2^ = 74%) & (MD = 1.06; 95% CI: −0.25, 2.38; *p* = 0.16; *χ* ^2^ = 3.71; *I* ^2^ = 46%) (Figure S1).

### 3.6 Results Intra‐Oral Spring Assisted Protraction

Only two studies were reported under this category which used 0.036‐in. beta‐nickel‐titanium helix spring assisted protraction with [43] and without TAD [58]. The force range for one study was 300g [43] while for the other it was 400–500 g [58]. Both studies used a double hinge type expander for the Alt‐RAMEC protocol, but one study used a 7‐week [43] and another used a 9‐week protocol for Alt‐RAMEC [58]. The quantitative synthesis could not be performed as angular dentoskeletal measurements were reported only in one study [43] and linear displacement of maxilla was not taken into account due to the risk of magnification errors.

### 3.7 Risk of Bias of Individual Studies

**FIGURE 4:**
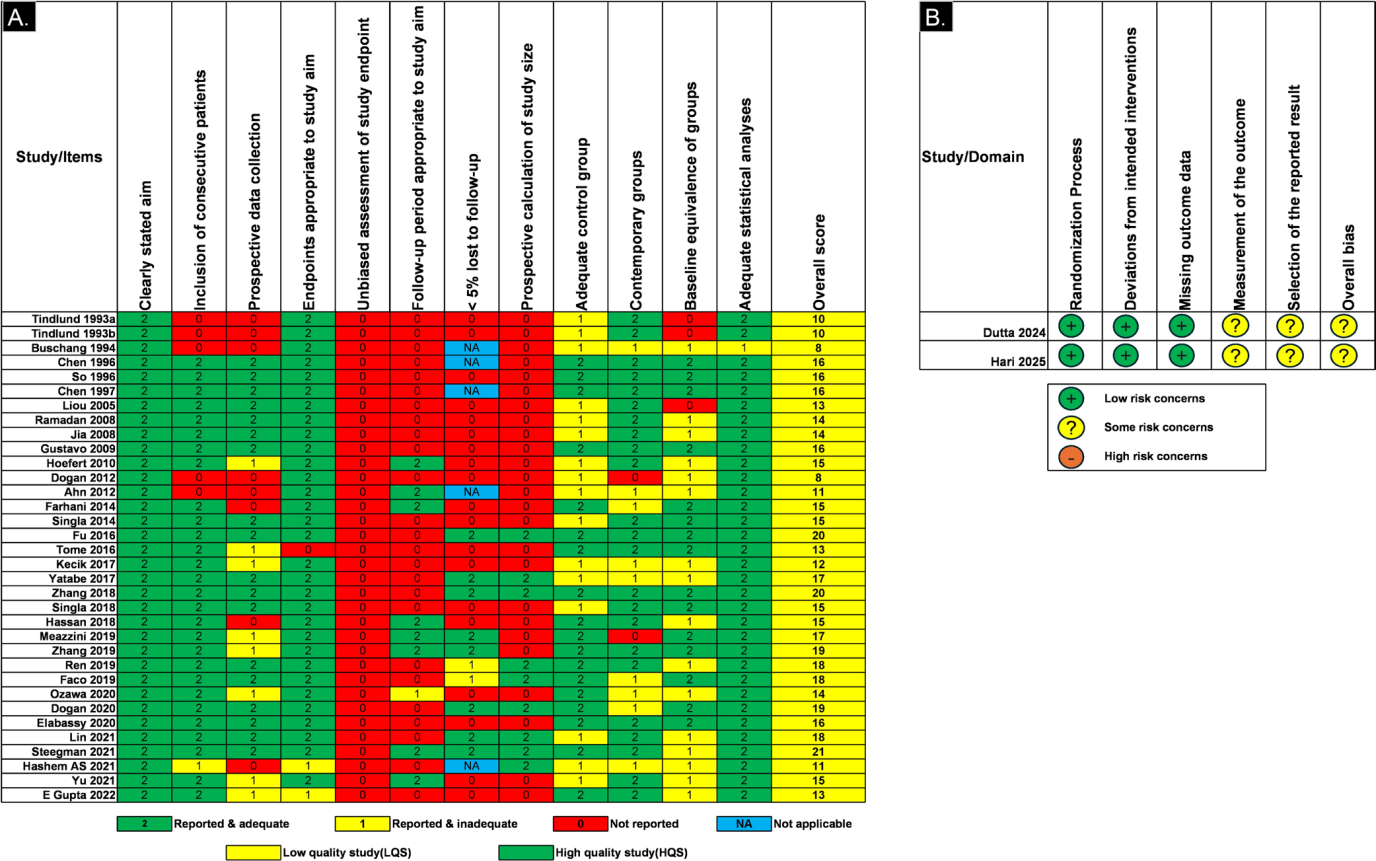
Risk of bias (A) MINORS (B) Cochrane RoB2 tool.

The MINORS (Methodological Index for Non‐Randomised Studies) assessment method was used to evaluate the quality of the eligible studies of the BES. Figure 4a lists included studies with its 12 criteria which were scored 0 (not reported), 1 (reported but inadequate), 2 (reported and adequate) depending on the information present in each article. The majority of the included literature had a high risk of bias as adequate information with regard to loss of follow‐up, sample size calculation, biased assessment of end‐point, and baseline equivalence of the control group was lacking. All non‐randomised studies scored below the cutoff point of 23, meaning all studies were assessed as LQS. The included RCTs [61, 62] were judged to be of overall low risk of bias but had some concerns in reported results and measurement of the outcome (Figure 4b).

### 3.8 Best Evidence Synthesis

A total of 36 studies were included in the BES process (Table 2). The synthesised evidence involved was based on subjects that underwent FM therapy, BAMP, TAD assisted protraction, spring assisted protraction, face mask‐miniplate (FMMP) treatment with or without RME/Alt‐RAMEC augmented with or without bone grafting procedures. Nineteen outcomes were reported in only one study and were therefore judged as insufficient evidence. Another five outcomes were reported in more than one study, out of which two pieces of evidence were regarded as moderate, which included P‐UCLAP versus NP‐UCLAP in FM therapy without expansion and P‐UCLAP versus non‐cleft because their data could be pooled together, while the remaining pool of three outcomes was deemed insufficient due to their low‐quality nature and inconsistency among studies.

**TABLE 2:** Results of best evidence synthesis.

Population	Synthesis of evidence	Level of scientific evidence	Origin of evidence	Study reference
Miniplate anchored facemask therapy				
UCLAP vs. BCLAP	UCLAP showed a more skeletal change compared to BCLAP	Insufficient	One study	Ahn [28] 2012
FMMP vs. FM in UCLAP	FMMP allows for a greater skeletal correction	Insufficient	One study	Dutta [61] 2024
FMMP in UCLAP	Despite long term use of FMMP therapy, 40% patient needs surgery	Insufficient	One study	Yu [36] 2021
Bone anchored maxillary protraction				
Td vs. Ut UCLAP	Significant improvement in maxillo‐mandibular relations in short‐term	Moderate	Two LQS	Faco [39] 2019, Ren [48] 2019
‐do—	Long‐term improvement in maxillo‐mandibular relations	Insufficient	One study	Steegman [46] 2021
Exp vs. no Exp UCLAP	No significant difference in maxillary advancement between groups	Insufficient	One study	Elabassy [60] 2020
Td UCLAP vs. Td non‐cleft	No significant difference in maxillary advancement	Insufficient	One study	Yatabe [55] 2017
Intra‐oral protraction spring				
Alt‐RAMEC vs. RME UCLAP	Alt‐RAMEC assisted expansion allowed greater maxillary advancement	Insufficient	One study	Liou [58] 2005
Td vs. Ut UCLAP	Alt‐RAMEC assisted expansion allowed greater maxillary advancement	Insufficient	One study	Meazzini [43] 2019
Conventional facemask therapy				
Td vs. Ut UCLAP	FM with Alt‐RAMEC protocol in late age groups still produces significantly forward movement in maxilla in comparison with the control group	Insufficient	One study	Farahani [29] 2014
	Short‐term improvement in GOSLON scores as compared to control	Insufficient	Two LQS	Ozawa [32] 2020, Hasan [42] 2018
	Significant skeletal & dentoalveolar improvement as compared to control	Moderate	Six LQS	Singla [44] 2014, So^82^ 1996, Chen [30] 1996, Fu [40] 2016 Ekta [41] 2022, Zhang [63] 2018
	Significant improvement in soft tissue profile as compared to control	Insufficient	One study	Chen [31] 1997
Td UCLAP vs. Td UCLAP	Favourable maxillary advancement in long term in patients with UCLP	Insufficient	One study	Zhang [57] 2019
Td UCLAP vs. Ut non cleft	RME + FM improves maxillary advancement in UCLAP	Insufficient	Two LQS	Buschang [11] 1994, Dogan [38] 2012
‐do—	RME + FM improves soft tissue profile in UCLAP	Insufficient	One study	Hashem AS [50] 2021
Td UCLAP vs. Td non‐cleft	Improves maxillo‐mandibular relation by pronounced effects in mandible in patients with UCLAP than non‐cleft	Moderate	Four LQS	Jia [51] 2008, Kecick [52] 2017, Lin [53] 2021, Ramadan [54] 2008
‐do—	The RME + FM improves soft tissue profile	Insufficient	One study	Sadehoefert [49] 2010
Alt‐RAMEC vs. RME UCLAP	No significant difference in maxillary advancement between the two protocols	Insufficient	One study	Gustavo [56] 2009
Alt‐RAMEC vs. no Exp UCLAP	Alt‐RAMEC protocol induced more skeletal, dentoalveolar and soft tissue changes in comparison to no Exp	Insufficient	One study	Dogan [37] 2020
‐do—	FM Alt‐RAMEC induces more skeletal changes than TransForce appliance	Insufficient	One study	Hari A V [62] 2025
UCLAP vs. BCLAP	Greater skeletal advancement of maxilla in UCLAP	Insufficient	One study	Tindlund [33] 1993a
‐do—	Soft tissue changes were similar in both groups	Insufficient	One study	Tindlund [34] 1993b
2‐stage vs. pushback palatoplasty UCLAP	Greater dentoskeletal improvement in Furlow technique than Pushback Palatoplasty technique	Insufficient	One study	Tome [35] 2016
SABG vs. No‐SABG UCLAP	Greater advancement of Point A in grafted group than non‐grafted	Insufficient	One study	Zhang [63] 2018

Abbreviations: Alt‐RAMEC, alternate rapid maxillary expansion and contraction; BCLAP, bilateral cleft lip, alveolus, and palate; Exp, expansion; FM, face mask; FMMP, face mask miniplate; RME, rapid maxillary expansion; SBAG, secondary alveolar cone graft; Td, treated; UCLAP, unilateral cleft lip, alveolus, and palate; Ut, untreated.

## 4 Discussion

Overall, this evidence synthesis and meta‐analysis shows that maxillary protraction is effective in moving the maxilla forward in patients with UCLAP in the short term, whether or not the protraction is combined with maxillary expansion. However, the level of evidence is weak to moderate. As per the evidence of maxillary protraction in Class III by FM therapy, it indicates the following parameters: 300–600 g force per side; force vector direction between 20° and 30° below the occlusal plane or parallel to the occlusal plane; and duration from 10 to 24 h of use per day [12, 65]. These values agree within the literature of the included studies of our review. However, the changes in SNA (2.64°), SNB (−1.9°) and ANB (4.34°) of P‐UCLAP versus NP‐UCLAP of our review were marginally more than those reported for Class III subjects versus untreated Class III (SNA = 1.75°, SNB = −1.78°, ANB = 3.64°) [65]. This could be due to greater pre‐existing maxillary retrognathia in repaired UCLAP participants [66]. In addition, when the P‐UCLAP group was compared with those treated Class III in our study, there was a more significant vertical increase in the UCLAP group. This could be because the scar tissue could hinder or even stop the vomer from gliding along the maxilla [67]. Hence, when the resistance of protraction is met, all the force might be directed to rotate the mandible and, consequently, increase the vertical.

The impact of expansion during maxillary protraction remains unclear due to limited high‐quality, comparable studies and similar controls. Two studies reported dentoskeletal parameters on the role of FM + RME in P‐UCLAP versus non‐cleft control, but one had Class I [11] and the other had Class III participants as control [38]. While another study reported their results of FM + RME assisted protraction on soft tissues in P‐UCLAP vs. treated non‐cleft control by 3‐Dimensional superimposition of anatomical points only [49]. The use of Alt‐RAMEC, as reported in a few studies [37, 58] has shown potential benefits when compared with untreated cohorts. However, it did not seem to provide an additional positive effect over maxillary protraction when compared with RME performed in P‐UCLAP [56]. Interestingly, similar results have been reported in a previously published reviews in the Class III population, where supplementing FM with RME [68] or Alt‐RAMEC seems to have minimal effect [69].

A previously published systematic review and meta‐analysis [12] comparing UCLAP with controls reported similar outcomes of improvement in SNA, SNB, and ANB. Compared to that review, our study included a larger number of studies and explored differences in facemask (FM) effects between individuals with UCLAP and the general Class III population. Also, in their paper, one study [63] was placed in the maxillary expansion group, which clearly mentioned that no expansion was conducted. Additionally, we examined the outcomes of BAMP and FMMP in UCLAP cases as well. We used the BES method. This approach clearly outlines different aspects of protraction therapy and offers an unbiased way to assess evidence, such as using RME versus Alt‐RAMEC. With regard to skeletally anchored protraction, the findings of a previously published review [20] are in lieu with ours about the qualitative effectiveness of BAMP and FMMP in reducing maxillary retrognathia. Nevertheless, they only evaluated results of the experimental group, which may induce overestimation of the effect size, limiting its generalisability [70].

With regards to the included studies, only publications that included cephalometric angular measurements were considered for the quantitative analysis in the current MA. Linear cephalometric measurements, in particular, are prone to magnification errors due to the nonparallel nature of X‐ray beams [71]. Although some studies have used cone‐beam computed tomography with a wide field of view to acquire cephalometric data, a recently published detailed methodological review has highlighted specific indications and dosage settings [72]. Furthermore, the diversity in the use of cephalometric landmarks among the included investigations adds another layer of complexity to the study. This diversity makes it challenging to appropriately evaluate and integrate the results of this study. The results from the BES have been outlined below.

### 4.1 UCLAP v/s BCLAP

Insufficient evidence by either technique used for maxillary protraction shows that more favourable changes are seen in the UCLAP population compared to BCLAP for skeletally anchored maxillary protraction [28] as well as FM assisted protraction therapy [33].

### 4.2 BAMP in UCLAP Population

Significant maxillary orthopaedic traction without dental side effects was shown in the experimental group in two low‐quality studies that constitute together some moderate evidence compared to the UCLAP controls [39, 48]. Thus, a bimaxillary miniplate could be instituted as an adequate orthopaedic maxillary traction in UCLP patients, but more studies are required for quantitative estimation.

Insufficient evidence from one study [60] shows no dentoskeletal improvements in combining the BAMP technique with maxillary expansion in UCLP patients, demonstrating that the added value of expansion is unclear.

### 4.3 FM v/s Miniplate‐Assisted FM Therapy

Only one study [61] was found under this cohort, so the evidence was graded inadequate.

### 4.4 Alt‐RAMEC v/s Conventional RME or No Expansion

The isolated evidence from results for Alt‐RAMEC v/s conventional RME in the included review states insufficient evidence, as one study [58] reported enhanced dentoskeletal effects on the use of Alt‐RAMEC while the other found no difference [56]. The results could not be meta‐analysed as the former used protraction springs and the latter employed FM. Two studies reported results comparing Alt‐RAMEC vs. no expansion in FM‐assisted protraction, with one study [29] having untreated control while the other had treated control [37]. Another study compared dentoskeletal effects of Alt‐RAMEC assisted FM with sagittal TransForce appliance [62]. However, all the evidence was deemed insufficient due to LQS or reduced number.

### 4.5 Conventional FM in UCLAP

#### 4.5.1 V/s Untreated UCLAP

The evidence under this section had the largest number of studies present in this group, but the majority of them were retrospective in nature, had inadequate information about loss to follow‐up, and prospective size calculations. Out of these, the results from six studies [10, 30, 40, 41, 44, 63] were pooled together, of which all of them were LQS. Overall, moderate evidence could be contemplated about the role of FM therapy in its effectiveness of maxillary protraction without expansion. Although the use of Alt‐RAMEC supported late maxillary protraction, no concrete evidence could be formulated. The effect over soft tissue was reported in detail by only one study [31] and therefore, it was graded as a piece of insufficient evidence.

#### 4.5.2 V/s Treated CIII (Non‐Cleft) Subjects Only

The included studies [51, 52, 53, 54] had non‐cleft Class III participants as their control subjects. SNA was marginally higher in the cleft group (*p* > 0.01). However, ANB changes were greater (*p* < 0.001) in UCLAP due to a greater decrease in SNB values of the cleft group. The studies did mention a brief period of expansion carried out before protraction, but details on the amount of expansion were not available. The synthesised evidence was graded as moderate as it included all LQS where the data could be pooled together.

#### 4.5.3 V/s Untreated Non‐Cleft Subjects

Results of multiple inconsistent studies [11, 38, 50] showed that FM was effective in maxillary protraction in UCLP when compared to untreated non‐cleft participants. However, due to inconsistencies in the methodology with respect to the classification of malocclusion of control subjects and the amount of expansion in study groups, it was deemed insufficient. One study [50] showed that using a hyrax expander and face mask resulted in considerable improvement in the facial soft tissue profile.

##### 4.5.3.1 SABG v/s Non‐SABG

There was greater advancement in point A and lesser mandibular backward rotation in the group that went bone grafting prior to maxillary protraction, as reported by a single study [63]. However, due to the lack a of more adequate studies reporting this result, it was labelled insufficient.

##### 4.5.3.2 Stability Assessment of MP by FM by GOSLON Yardstick Index

To assess the improvement in the dental arch relationship, few studies use the GOSLON yardstick. Only two studies [32, 42] were found in this cohort. Use of FM for MP provided satisfactory rapid results, outperforming the untreated sample despite their instability. However, the synthesised evidence was insufficient as the data could not be pooled due to the qualitative nature of the experiment. Moreover, the participants in one study also underwent maxillary expansion [32].

##### 4.5.3.3 Technique of Hard Palate Repair

Only one study on 19 patients [35] evaluated the dentoskeletal comparison based on the difference in the type of hard palate repair. Maxillary protraction was more effectively achieved in UCLP patients after early two‐stage palatoplasty than after push‐back palatoplasty.

## 5 Limitations

Only two RCT trials could be identified for this review [61, 62]. Most studies were classified as low quality studies. Furthermore, significant variability exists in the methodologies employed across studies, including differences in imaging techniques, measurement standards and sampling characteristics. These discrepancies complicate direct comparisons and meta‐analyses. The primary limitation identified in the selected studies was the absence of randomisation. This may stem from the challenges associated with conducting a randomised controlled trial (RCT) that involves extended follow‐up and ethical concerns [73]. Long‐term effect until growth has ceased was also not considered in the studies. Additionally, the variability in surgical repair techniques for cleft conditions is a limitation, as different methods may lead to varying levels of scarring, which in turn can affect maxillary growth disturbances [74]. The extent and severity of the cleft type affect craniofacial morphology [75], but none of the studies reported specific characteristics. Recent literature suggests that the laterality (right or left sidedness) of the cleft may be linked to the severity of the phenotype and associated anatomical anomalies [76, 77]. Furthermore, none of the studies reported on adverse effects and patient‐reported outcomes, and patient‐reported experiences were also not included (PROs and PREs) [78].

## 6 Future Recommendations

High‐quality randomised controlled trials (RCTs) should be the primary focus of future research on maxillary protraction for UCLAP patients. To better understand the actual therapy effect, research comparing the results of UCLAP patients treated with and without specific protraction techniques is needed. The most effective protocol must also be established by conducting head‐to‐head RCTs comparing various therapeutic modalities, such as rapid maxillary expansion (RME) versus alternate RME and constriction (Alt‐RAMEC) or traditional facemask therapy versus bone‐anchored maxillary protraction (BAMP). These studies should use adequately powered sample sizes to assure generalisability. Furthermore, long‐term follow‐up that continues until maxillofacial growth stops is required to assess the stability and durability of therapy outcomes, which are currently lacking in the research.

Future studies should also use three‐dimensional (3D) imaging methods to improve diagnostic accuracy. Cone‐beam computed tomography (CBCT) at ultra‐low doses may be a viable alternative since it balances the necessity of a thorough skeletal examination and the requirement to reduce radiation exposure in growing patients [79]. The 3D analysis would contribute to evidence‐based, patient‐centred therapy in UCLAP management by offering a more thorough understanding of skeletal, dental, and soft tissue changes [80].

## 7 Conclusion

Within the limitations of the current review, FM therapy seems to have a positive influence on the maxillofacial growth of UCLAP in the short term, as reflected in dentoskeletal parameters. The supplementary role of expansion could not be explored due to the unavailability of data and the limited number of studies. The results from low‐quality studies on skeletally anchored protraction suggest that BAMP or FM‐MP could be a potential option because of a more skeletal response, but the evidence remains weak due to a low number of studies. Additionally, no evidence for expansion along with BAMP could be corroborated. We recommend that future studies conducted with more scientific rigour should include long‐term follow‐up, ideally until maxillofacial growth is finished, to evaluate skeletal, dental and soft tissue changes over the short and long term.

## Author Contributions

Idea: Karthik Sennimalai and Hamza Parvez Siddiqui; Literature search: Hamza Parvez Siddiqui, Jitender Machawal and Karthik Sennimalai; Formal analysis and investigation: Karthik Sennimalai, Hamza Parvez Siddiqui and Anne Marie Kuijpers‐Jagtman; Writing – original draft preparation: Hamza Parvez Siddiqui; Writing – review and editing: Hamza Parvez Siddiqui, Anne Marie Kuijpers‐Jagtman, Maria C. Meazzini, Madhanraj Selvaraj; Supervision: Anne Marie Kuijpers‐Jagtman.

## Conflicts of Interest

The authors declare no conflicts of interest.

## Supporting information


Data S1.

Data S2.

Data S3.

## Data Availability

The necessary datasets used and analysed during the current study are available from the corresponding author on a reasonable request.
