# Supplementary figures and images for: Effects of Maxillary Protraction Techniques on Maxillofacial, Dental, and Soft Tissue Outcomes in Patients With Non‐Syndromic Unilateral Cleft Lip and Palate: A Systematic Review and Meta‐Analysis
Source: Orthod Craniofac Res. 2025 Aug 8;28(6):885–906. doi: 10.1111/ocr.70014 (PMC12603673; doi:10.1111/ocr.70014)

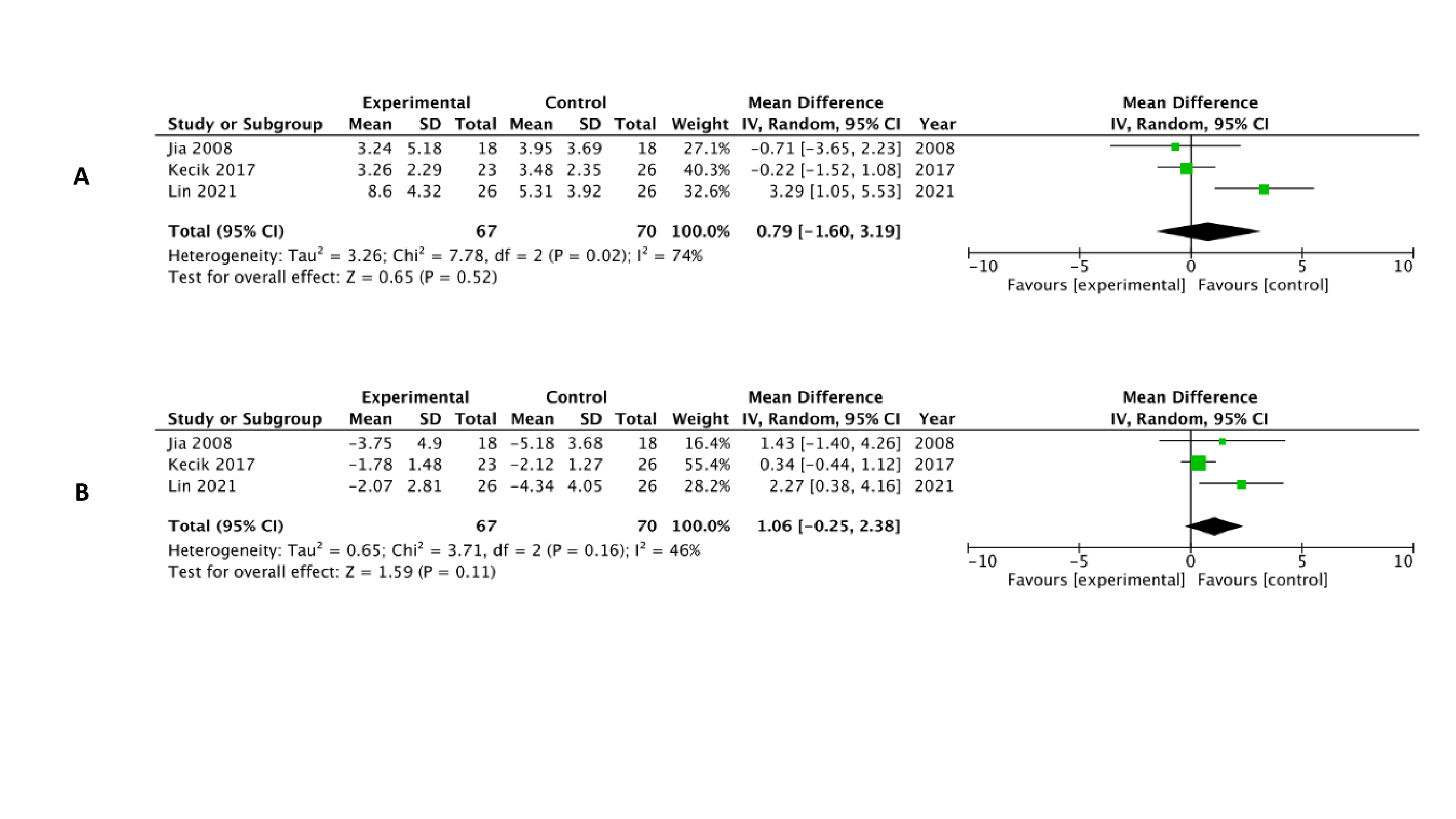

Supplement: Supplementary file 1 — Data S1. [file OCR-28-885-s002.tiff]

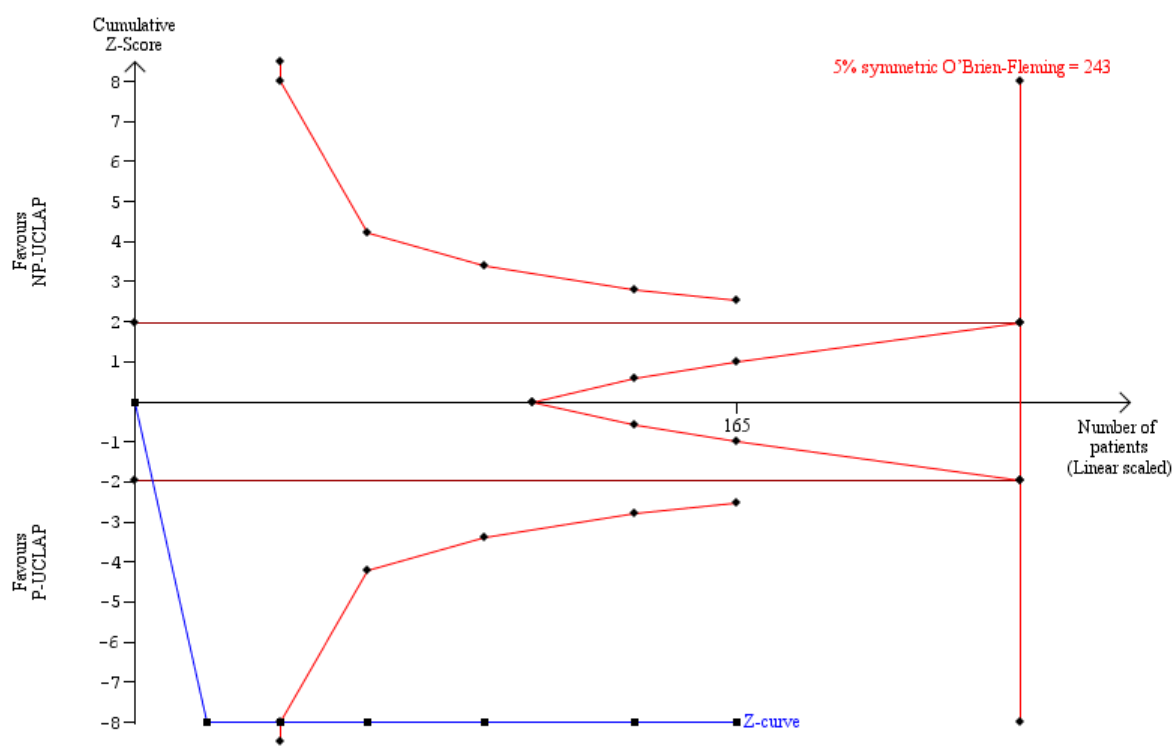

Supplement: Supplementary file 2 — Data S2. [file OCR-28-885-s003.tiff]
