# Supplementary material for: Effects of Maxillary Protraction Techniques on Maxillofacial, Dental, and Soft Tissue Outcomes in Patients With Non‐Syndromic Unilateral Cleft Lip and Palate: A Systematic Review and Meta‐Analysis
Source: Orthod Craniofac Res. 2025 Aug 8;28(6):885–906. doi: 10.1111/ocr.70014 (PMC12603673; doi:10.1111/ocr.70014)
Supplement: Supplementary file 3 — Data S3. [file OCR-28-885-s001.docx]

Supplementary Table s1: Search strategy for databases

| Database | Search string | Articles |
| --- | --- | --- |
| PubMed | ("Cleft Lip"[Mesh] OR "Cleft Palate"[Mesh] OR "orofacial cleft" OR cleft OR "cleft lip and palate" OR "cleft lip and/or palate" OR "cleft lip" OR "cleft palate" OR "cleft lip palate") AND ("Malocclusion, Angle Class III"[Mesh] OR “Angle Class III” OR “Class III malocclusion” OR “skeletal class III” OR "midface deficiency" OR “maxillary deficiency” OR “retrognathic maxilla” OR “maxillary retrognathism” OR “maxillary hypoplasia” OR “hypoplastic maxilla”) AND (protraction OR traction OR advancement OR facemask OR “face mask” OR “reverse pull headgear” OR “protraction headgear” OR Delaire OR Petit OR "Orthodontic Anchorage Procedures"[Mesh] OR “Bone anchored maxillary protraction” OR BAMP OR “Bone anchor” OR “Skeletal anchor” OR “Mini plate” OR Miniplate OR “Bone borne” OR Bone-borne OR “Mini screw” OR Miniscrew OR "micro screw" OR microscrew OR Microimplant OR “Mini implant” OR “Micro implant” OR Microimplant OR “Temporary anchorage device” OR TAD) | 320 |
| Scopus | ( TITLE-ABS-KEY ( "orofacial cleft" OR cleft OR "cleft lip and palate" OR "cleft lip and/or palate" OR "cleft lip" OR "cleft palate" OR "cleft lip palate" ) AND TITLE-ABS-KEY ( "Angle Class III" OR "Class III malocclusion" OR "skeletal class III" OR "midface deficiency" OR "maxillary deficiency" OR "retrognathic maxilla" OR "maxillary retrognathism" OR "maxillary hypoplasia" OR "hypoplastic maxilla" ) AND TITLE-ABS-KEY ( protraction OR traction OR advancement OR facemask OR "face mask" OR "reverse pull headgear" OR "protraction headgear" OR delaire OR petit OR "Bone anchored maxillary protraction" OR bamp OR "Bone anchor" OR "Skeletal anchor" OR "Mini plate" OR miniplate OR "Bone borne" OR bone-borne OR "Mini screw" OR miniscrew OR "micro screw" OR microscrew OR microimplant OR "Mini implant" OR "Micro implant" OR microimplant OR "Temporary anchorage device" OR tad ) ) | 338 |
| Embase | (('orofacial cleft' OR cleft OR 'cleft lip and/or palate' OR 'cleft lip' OR 'cleft palate' OR 'cleft lip palate'):ti,ab,kw AND ('angle class iii' OR 'class iii malocclusion' OR 'skeletal class iii' OR 'midface deficiency' OR 'maxillary deficiency' OR 'retrognathic maxilla' OR 'maxillary retrognathism' OR 'maxillary hypoplasia' OR 'hypoplastic maxilla'):ti,ab,kw AND (protraction OR traction OR advancement OR facemask OR 'face mask' OR 'reverse pull headgear' OR 'protraction headgear' OR delaire OR petit OR 'bone anchored maxillary protraction' OR bamp OR 'bone anchor' OR 'skeletal anchor' OR 'mini plate' OR miniplate OR 'bone borne' OR 'mini screw' OR miniscrew OR 'micro screw' OR microscrew OR 'mini implant' OR 'micro implant' OR microimplant OR 'temporary anchorage device' OR tad):ti,ab,kw | 295 |
| Web of Science (All databases) | Cleft (Topic) and (“Angle Class III” OR “Class III malocclusion” OR “skeletal class III” OR "midface deficiency" OR “maxillary deficiency” OR “retrognathic maxilla” OR “maxillary retrognathism” OR “maxillary hypoplasia” OR “hypoplastic maxilla”) (Topic) and (protraction OR traction OR advancement OR facemask OR “face mask” OR “reverse pull headgear” OR “protraction headgear” OR delaire OR Petit OR “Bone anchored maxillary protraction” OR bump OR “Bone anchor” OR “Skeletal anchor” OR “Mini plate” OR manipulate OR “Bone borne” OR Bone-borne OR “Mini screw” OR miniscrews OR "micro screw" OR microscrews OR microimplant OR “Mini implant” OR “Micro implant” OR microimplant OR “Temporary anchorage device” OR TAD) (Topic) | 473 |
| EBSCOhost | (cleft and ("Angle Class III" or "Class III malocclusion" or "skeletal class III" or "midface deficiency" or "maxillary deficiency" or "retrognathic maxilla" or "maxillary retrognathism" or "maxillary hypoplasia" or "hypoplastic maxilla") and (protraction or traction or advancement or facemask or "face mask" or "reverse pull headgear" or "protraction headgear" or delmaire or Petit or "Bone anchored maxillary protraction" or bump or "Bone anchor" or "Skeletal anchor" or "Mini plate" or Miniplate or "Bone borne" or Bone-borne or "Mini screw" or Miniscrew or "micro screw" or microscrews or microimplants or "Mini implant" or "Micro implant" or microimplants or "Temporary anchorage device" or TAD)) | 121 |
| Ovid | (cleft and ("Angle Class III" or "Class III malocclusion" or "skeletal class III" or "midface deficiency" or "maxillary deficiency" or "retrognathic maxilla" or "maxillary retrognathism" or "maxillary hypoplasia" or "hypoplastic maxilla") and (protraction or traction or advancement or facemask or "face mask" or "reverse pull headgear" or "protraction headgear" or delmaire or Petit or "Bone anchored maxillary protraction" or bump or "Bone anchor" or "Skeletal anchor" or "Mini plate" or Miniplate or "Bone borne" or Bone-borne or "Mini screw" or Miniscrew or "micro screw" or microscrews or microimplants or "Mini implant" or "Micro implant" or microimplants or "Temporary anchorage device" or TAD)).ti,ab,kw. | 81 |
| LILACS | ("orofacial cleft" OR cleft OR "cleft lip and palate" OR "cleft lip and/or palate" OR "cleft lip" OR "cleft palate" OR "cleft lip palate") AND (“Angle Class III” OR “Class III malocclusion” OR “skeletal class III” OR "midface deficiency" OR “maxillary deficiency” OR “retrognathic maxilla” OR “maxillary retrognathism” OR “maxillary hypoplasia” OR “hypoplastic maxilla”) AND (protraction OR traction OR advancement OR facemask OR “face mask” OR “reverse pull headgear” OR “protraction headgear” OR Delaire OR Petit OR “Bone anchored maxillary protraction” OR BAMP OR “Bone anchor” OR “Skeletal anchor” OR “Mini plate” OR Miniplate OR “Bone borne” OR Bone-borne OR “Mini screw” OR Miniscrew OR "micro screw" OR microscrew OR Microimplant OR “Mini implant” OR “Micro implant” OR Microimplant OR “Temporary anchorage device” OR TAD)) in Title, Abstract, Subject | 39 |
| Cochrane  Library | (("orofacial cleft" OR cleft OR "cleft lip and palate" OR "cleft lip and/or palate" OR "cleft lip" OR "cleft palate" OR "cleft lip palate") AND (“Angle Class III” OR “Class III malocclusion” OR “skeletal class III” OR "midface deficiency" OR “maxillary deficiency” OR “retrognathic maxilla” OR “maxillary retrognathism” OR “maxillary hypoplasia” OR “hypoplastic maxilla”) AND (protraction OR traction OR advancement OR facemask OR “face mask” OR “reverse pull headgear” OR “protraction headgear” OR Delaire OR Petit OR “Bone anchored maxillary protraction” OR BAMP OR “Bone anchor” OR “Skeletal anchor” OR “Mini plate” OR Miniplate OR “Bone borne” OR Bone-borne OR “Mini screw” OR Miniscrew OR "micro screw" OR microscrew OR Microimplant OR “Mini implant” OR “Micro implant” OR Microimplant OR “Temporary anchorage device” OR TAD)) in Title Abstract Keyword - (Word variations have been searched) | 25 |
| Google Scholar | Bone Anchored Maxillary Protraction/ Face mask assisted maxillary protraction in Unilateral Cleft lip & Palate | 200 |
